# “Thoughts and Health”, the future prevention for adolescents today - Preventing a first depression

**DOI:** 10.1192/j.eurpsy.2022.1069

**Published:** 2022-09-01

**Authors:** C. Wikberg, P. Augustsson, G. Sveinsdottir, E.Ö. Arnarsson, E. Craighead, I. Marteinsdottir, J. Lilja

**Affiliations:** 1 Medicine - University of Gothenburg, School Of Public Health And Community Medicine, Gothenburg, Sweden; 2 School of Health Sciences, University of Iceland, Faculty Of Medicine, Reykjavík, Iceland; 3 Medicine, School Of Health Sciences, Reykjavik, Iceland; 4 Psychology, Department Of Psychiatry And Behavioral Sciences, Georgia, United States of America; 5 Medicine and Optometry, Faculty Of Health And Life Sciences, Kalmar, Sweden; 6 Psychology, Department Of Psychology, Gothenburg, Sweden

**Keywords:** Depression, prevention, Adolescense

## Abstract

**Introduction:**

Mental illness is a growing problem among adolescents. Adolescents are sensitive and at increased risk of developing a first depression. There are knowledge gaps about the long-term effects of prevention programs against depresson for adolescents.

**Objectives:**

A randomized controlled study among Swedish adolescents in eighth grade, who are at risk of developing depression. The study examines the long-term effects of the “Thoughts and Health” prevention program and whether it is as effective Online as In Real Life (IRL).

**Methods:**

In a first step, about 20 junior high schools in the Västra Götaland region will be recruited and randomised into one of three groups.

The adolescents are screened for depression at school

Group 1 - Adolescents at risk of developing depression receive the course program “Thoughts and Health” Online.

Group 2 - Adolescents at risk of developing depression receive the course program “Thoughts and Health” IRL.

Group 3 - Adolescents at risk of developing depression receive the usual school health care (control group).

Psychologists decide inclusion after a diagnostic interview. OUTCOME VARIABLES

Quantitative

- development of depression is measured via self-assessment instruments and follow-up assessment, by a psychologist.

- school attendance and full grades at the end of compulsory school.

- biomarkers

Qualitative

Adolescent’s experiences and perceptions of the course program.

**Results:**

Will create evidence for prevention programs against depression and be used to develop primary prevention for adolescents Online and IRL, which will be of great importance to public health.

**Conclusions:**

Thoughts and Health can be a useful tool to prevent depression among adolescents

**Disclosure:**

No significant relationships.

